# Polymorph-Dependent Photophysics of Blue-Emitting Brominated Organic Crystals

**DOI:** 10.1021/acsaom.6c00105

**Published:** 2026-06-15

**Authors:** Haydee Pacheco, Jesus Valdiviezo, Rianne G. De Leon, Thomas J. Emge, Mircea Cotlet, Deirdre M. O’Carroll

**Affiliations:** † Department of Materials Science and Engineering, 151434 Rutgers University, 607 Taylor Road, Piscataway, New Jersey 08854, United States; ‡ Department of Biological Chemistry and Molecular Pharmacology, 1811 Harvard Medical School, Boston, Massachusetts 02115, United States; § Department of Cancer Biology, Dana-Farber Cancer Institute, Boston, Massachusetts 02215, United States; ∥ Sección Química, Departamento de Ciencias, Pontificia Universidad Católica del Perú, San Miguel, Lima 15088, Peru; ⊥ Center for Functional Nanomaterials, 8099 Brookhaven National Laboratory, Upton, New York 11973, United States; # Department of Chemistry and Chemical Biology, Rutgers University, 123 Bevier Road, Piscataway, New Jersey 08854, United States

**Keywords:** room-temperature phosphorescence, organic polymorphism, excitonic coupling, halogen bonding, J-aggregates, photoluminescence, microcrystals

## Abstract

Nonmetal-containing organic molecules typically do not emit blue phosphorescence efficiently due to weak intersystem crossing and the challenge of protecting high-energy triplet states from nonradiative decay. We show that use of triplet-promoting bromine atoms and careful control of molecular packing can lead to well-defined, efficient room-temperature phosphorescence (RTP) from organic crystals. We report on the distinct optical properties of a polymorphic bromine-containing organic molecule, 1,4-bis­(bromomethyl)-2,5-bis­(octyloxy)­benzene (Br8) in solid-state (micro)­crystal and thin-film forms. The two polymorphs, named Br8-H and Br8-J, exhibit different molecular packing arrangements in single crystals and thin films and, consequently, different photophysical behaviors. The Br8-J polymorph is characterized by a head-to-tail molecular arrangement in a triclinic crystal, while the Br8-H polymorph adopts a face-to-face arrangement (monoclinic crystal). Br8-J has a higher quantum yield (38% for the microcrystal form and 8% for the thin film), lower energy, and narrower emission, compared to Br8-H (quantum yield of just 2% for the microcrystal and 0.2% in the thin film). The higher quantum yields in the microcrystal forms indicate the key role of intermolecular interactions in reducing nonradiative recombination in these polymorphs. The presence of both prompt and delayed blue emission (in the range 420–470 nm) and large Stokes shifts from both polymorphs indicates the involvement of triplet excited states and the occurrence of RTP with lifetimes of ∼325 μs. With an emission wavelength of 470 nm and a quantum yield of 38%, the Br8-J polymorph demonstrates how specific head-to-tail packing can unlock highly efficient solid-state phosphorescence compared to its face-to-face counterpart. The optimal intermolecular halide bonding in the J-type packing arrangement combined with the heavy atom effect is key to unlocking efficient blue emission from Br8 crystals. Our findings uncover rich photophysics in halogenated organic molecules in the solid state and the importance of molecular packing in their emission characteristics.

## Graphical abstract

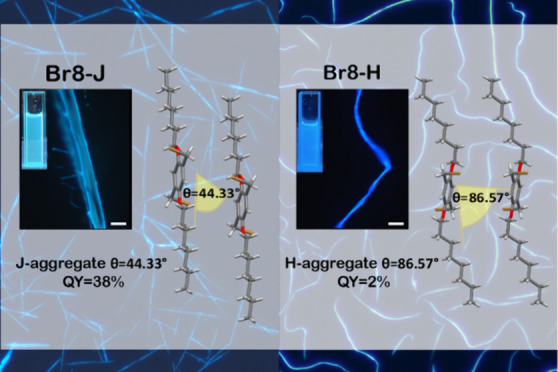

## Introduction

Purely organic light-emitting materials have emerged as a promising frontier in the field of organic electronics offering versatility in molecular design and engineering. Light emission from these materials proceeds by either fluorescence or phosphorescence with the latter typically being highly inefficient due to the spin statistics of the triplet state.^1–3^ However, numerous strategies have been introduced to allow phosphorescence emission from the triplet state to overcome the limitations of spin statistics and achieve efficient phosphorescence from organic materials.^1,4^ Typically, heavy metal atoms like iridium, platinum and copper are used to enhance spin–orbit coupling (SOC), facilitating intersystem crossing (ISC) between singlet and triplet states. This process promotes efficient phosphorescence by increasing triplet state population and deactivating nonradiative decay pathways.^5–12^ While reported organic molecules usually have rather slow phosphorescence rates, between 10^–3^ and 10^3^ s^–1^, and nonradiative pathways prevail at room temperature,^13,14^ more recently, the design of organic phosphorescent materials has been further advanced by utilizing the concept of hybridized local and charge-transfer (HLCT) states. HLCT states, which combine the features of locally excited (LE) and charge-transfer (CT) states, can enhance SOC and facilitate ISC, leading to efficient room-temperature phosphorescence (RTP) by manipulating the electronic structure and spatial overlap of the involved orbitals.^3,15–18^ This approach can be used both with and without the addition of heavy atoms. RTP has also been demonstrated from organic molecules that incorporate nonmetal heavy atoms such as halides that have benefits due to their relative abundance and more stable bonding.^19–21^ Similar to heavy metals, they enhance SOC and suppress nonradiative decay, boosting phosphorescence efficiency.

Beyond individual molecules, crystal packing and polymorphism play crucial roles in determining photophysical properties in the solid state. Structural modifications, packing and crystal polymorphism in solid-state organic materials have been demonstrated to tune the singlet–triplet energy gap and chromophore stacking, favoring either thermally activated delayed fluorescence (TADF) or RTP.^3,22–24^ Polymorphs of the same molecule may exhibit either TADF or RTP with unique emission colors and efficiencies due to differences in intermolecular interactions and electronic environments.^23^ Different packing arrangements can significantly influence the material’s photophysical properties, such as emission wavelength, mechanism, quantum yield, and stability.^23,25^ In some cases, organic compounds have been found to exhibit both TADF and RTP concurrently.^26^ It has been reported that a hidden RTP state of a TADF molecule can potentially serve as a ‘springboard’ for long-lived triplet excitons and facilitate reverse intersystem crossing (RISC).^27^ The nature of molecular packing also plays a significant role in dictating emission behavior. J-type aggregates, characterized by head-to-tail molecular packing, often favor red-shifted, narrow-band emission due to enhanced excitonic coupling, whereas H-type aggregates, with face-to-face stacking, typically suppress radiative decay, leading to broader and blue-shifted emission.^28^ These aggregation effects can directly influence triplet-state populations, modifying the extent of ISC and RISC.

In this work, we demonstrate how distinct polymorphic packing arrangements in a bromine-containing organic molecule, 1,4-bis­(bromomethyl)-2,5-bis­(octyloxy)­benzene (Br8), lead to the emergence of deep-blue RTP. We show that differences in J-type versus H-type molecular packing within these polymorphs play a critical role in determining triplet-state emission efficiency and dynamics, shedding light on how molecular stacking can be leveraged to control RTP without extensive synthetic modifications. Small differences in packing arrangement significantly influence the material’s photophysical properties, such as absorption and emission wavelengths, Stokes shift, photoluminescence quantum yield (PLQY), and photostability. The controlled fabrication of the two distinct crystalline polymorphs of Br8, designated Br8-J and Br8-H, leads to RTP characteristics in solid state microcrystals and spin-coated thin films. The RTP quantum yields (Φ_P_) could be manipulated (from 8% to 38%) in microcrystals, while in the thin film, Φ_P_ only reaches 2%. Furthermore, the temperature dependence of the emission is drastically different between the polymorphs. This detailed investigation provides understanding of the phase-dependent RTP phenomena of halogenated organic solids.

## Results and Discussion

To study the photophysics of Br8 in the solid state, dispersions of Br8 crystals of the two different polymorphs were prepared using a controlled crystallization process, and detailed optical imaging and spectroscopy was employed (see Methods). The crystallization process was performed using an electron-withdrawing solvent (2-methoxyethanol), strategically chosen to optimize the molecular packing and favor the formation of highly ordered crystal structures. The crystallization was achieved through tailored solvent-based crystallization methods. Typically, Br8-J was synthesized by dissolving 4 mg of Br8 in 1 mL of 2-methoxyethanol, followed by alternating cycles of sonication and heating at 85 °C until a clear solution formed. Slow cooling (∼10 °C/h) facilitated the growth of needle-like crystals. In contrast, Br8-H was fabricated using the same concentration and solvent but included stirring at 500 rpm during the sonication-heating cycles, followed by rapid cooling to 7 °C, yielding thread-like crystals.

The optical and spectroscopic properties of each sample were investigated, and measurements were performed under ambient conditions unless otherwise specified. Figure 1 presents a comprehensive characterization of the Br8-J and Br8-H microcrystal dispersions in 2-methoxyethanol, single-crystal forms, and spin-coated thin films. The Br8-J microcrystals exhibit intense blue photoluminescence (PL) emission under an excitation wavelength of 365 nm (Figure 1b). The dimensions for both Br8-J and Br8-H are similar, approximately 50 μm in length and 100–500 nm in diameter. The morphology of the Br8-J crystals is rigid and needle-like (Figure 1a,b). For Br8-H microcrystals, the morphology and appearance are noticeably different. The dark-field scattering images are also blue, but the morphology is more thread-like and twisted compared to Br8-J microcrystals (Figure 1c), suggesting a different internal molecular packing. Additionally, there is negligible PL emission apparent from Br8-H under 365 nm excitation at room temperature (Figure 1d). The visual appearance of the Br8-J and Br8-H microcrystal dispersions prior to deposition are also clearly different, with Br8-J dispersions deep yellow in color and exhibiting light-blue emission under <350 nm UV lamp excitation (Figure 1a,b, insets), and Br8-H dispersions are light yellow in color with deep-blue emission under <350 nm excitation (Figure 1c,d, insets). This suggests that the Br8-H is in fact luminescent but has a higher bandgap compared to Br8-J. When spun into polycrystalline thin films using an antisolvent (see Methods), Br8-J and Br8-H show light-blue and deep-blue emission colors (Figure 1e,f) that are consistent with the microcrystal dispersions. The emission of both Br8-J and Br8-H was photostable under the excitation conditions employed for the duration of the luminescence measurements when encapsulated in optical epoxy (Figure S1).

**Figure 1:**
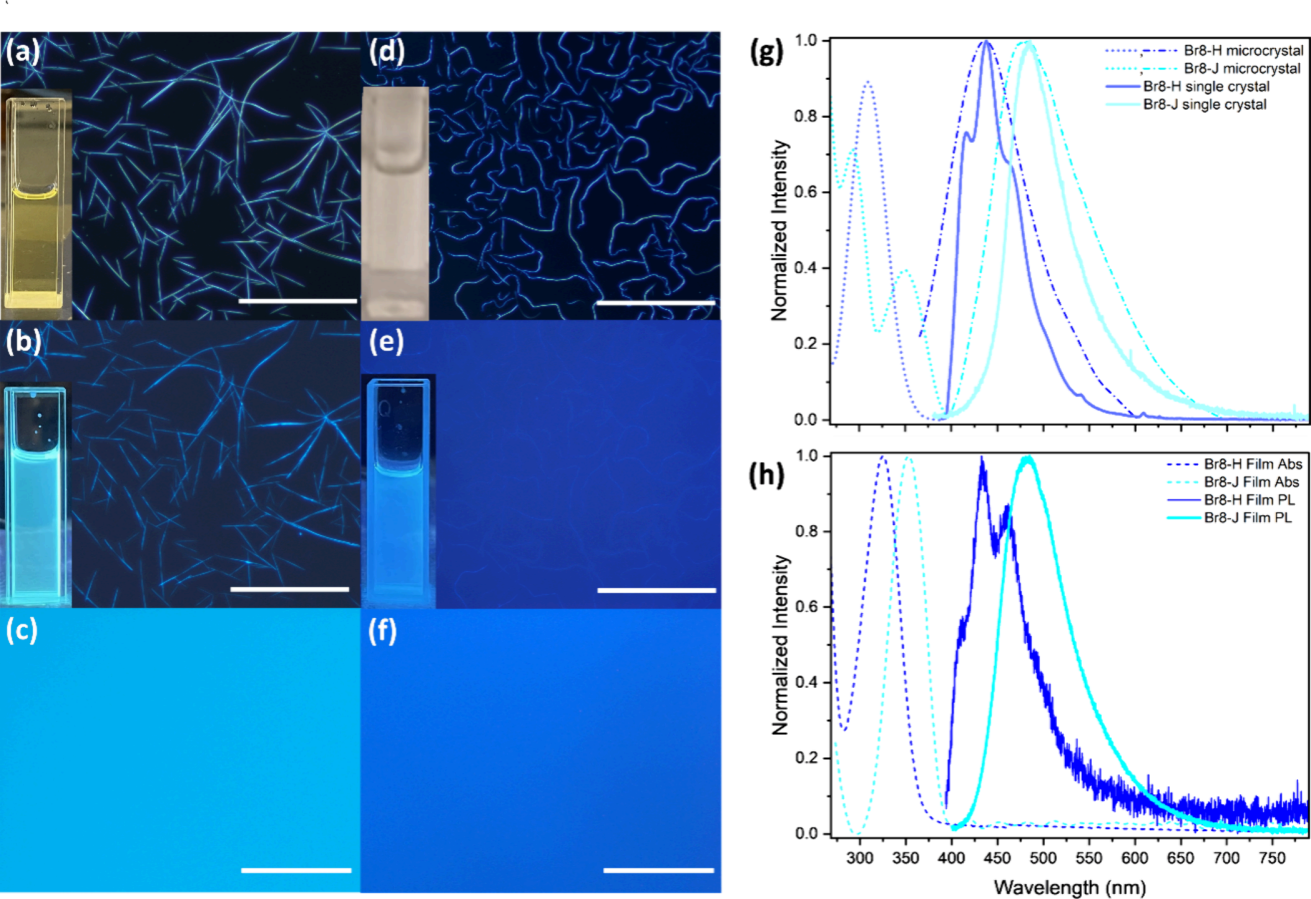
Optical and spectroscopic analysis of two polymorphs of Br8. (a and b) Dark-field and PL optical images of needle-like crystals of Br8-J spin-coated on glass, respectively (the scale bar is 50 μm). (d and e) Dark-field and PL optical images of thread-like crystals of Br8-H spin-coated on glass, respectively. An excitation wavelength of 365 nm was used for PL images in parts a and c (the scale bar is 50 μm). The main microscopy images in parts a, b, d, and e display the microcrystals after being drop-casted onto a glass substrate and dried under ambient conditions. The insets in parts a, b, d, and e are photographs of Br8-J and Br8-H microcrystal dispersions (4 mg/mL in 2-methoxyethanol; the scale bar is 200 μm) under ambient light, or 365 nm excitation. (c and f) PL images of the spin-coated thin films of Br8-J and Br8-H, respectively (the scale bar is 50 μm). The uniform PL profiles confirm the high morphological quality and macroscopic emission homogeneity of the films. (g) Absorption (dashed lines) and PL (dot-dash-dotted lines) spectra of Br8-J and Br8-H crystal dispersions (diluted to 0.1 mg/mL) and single-crystal PL spectra for Br8-J and Br8-H (solid lines). The dispersion samples are in 2-methoxyethanol. (h) Absorption (dashed lines) and PL (solid lines) spectra of Br8-J and Br8-H thin films. All measurements were performed under ambient conditions. For PL images, exposure times were autoscaled for optimal brightness and true-color detection.

Figure 1g shows the characteristic absorption and emission spectra of microcrystal dispersions and individual crystals of the two polymorphs. Br8-J exhibits absorption in two distinct bands: a higher-energy transition at 300 nm characteristic of a localized π → π* transition on the aromatic core, which typically requires higher excitation energy and is relatively insensitive to intermolecular packing. We have assigned the lower-energy band at 350 nm arises from a more extended, hybridized π → π* state. The dense solid-state packing induces significant mixing between the aromatic π system and the nonbonding orbitals of the heavy bromine atoms. This extended orbital delocalization lowers the required excitation energy (shifting the peak to 350 nm) and renders this highly mixed transition extremely sensitive to the specific J-type versus H-type crystallographic architectures. Supported by our solvatochromic analysis (Figure S9), this 350 nm band is assigned to a strongly allowed π → π* transition that is heavily hybridized with the bromine lone pairs due to dense intermolecular packing. In contrast, Br8-H displays a single, broader absorption band centered at 312 nm, which is similarly attributed to a hybridized π → π* state dictated by its distinct face-to-face molecular arrangement. The Stokes shifts are large (>115 nm) for both polymorphs. Br8-J PL emission occurs at 467 nm with a full-width at half-maximum (fwhm) of 118 nm for microcrystals and 67 nm for single crystals. Br8-H exhibits a narrower emission that occurs at 438 nm with a fwhm of 98 nm for microcrystals and 65 nm for single crystals. The single crystals of both polymorphs exhibit narrower PL emission spectra compared to the microcrystal dispersions due to less inhomogeneous broadening. The optical energy gaps, estimated from the low-energy onset of the solid-state absorption spectra, were determined to be 2.78 eV for Br8-J and 3.46 eV for Br8-H (Figure S2). PLQY values were 38% for Br8-J microcrystal dispersion samples under 365 nm excitation but decreased significantly to 2% for Br8-H. In the polycrystalline thin films, the Br8-J absorption and PL peak positions do not change significantly, while there is a notable reduction in the Stokes shift for Br8-H (Figure 1h), the quantum yield drops to 8% for Br8-J and 0.2% for Br8-H in these films (Table S7). These data indicate that the microcrystalline forms lead to less nonradiative recombination, likely due to restricted molecular motion, minimal lattice vibrations and fewer intermolecular interactions compared to the polycrystalline thin films.^29^ Consequently, these materials are more luminescent in the crystalline form and their emission behavior is dependent on the molecular packing arrangement in the crystal. Therefore, the highly ordered crystalline environment is crucial for efficient luminescence in the Br8 system. Rather than quenching the emission, the dense intermolecular interactions (such as halogen bonding) present in the single crystal serve to rigidify the molecular conformation, thereby suppressing nonradiative decay pathways and activating RTP. This is further evidenced by comparing the highly ordered microcrystals to the spin-coated thin films. The thin films exhibit a polycrystalline morphology characterized by a lack of long-range crystalline order. This increased structural disorder and the presence of defect states at the grain boundaries reintroduce nonradiative recombination pathways.

The examination of temperature-dependent PL spectra and PL lifetime measurements provides critical insights into the excitonic dynamics and emission mechanisms of polymorphs Br8-J and Br8-H (see Methods, Figure S3, Tables S1 and S2, and Figure 2a,b, insets). Efficient luminescence from Br8-H is only recovered at lower temperatures (e.g., <175 K) when thermally activated nonradiative quenching is suppressed. This temperature-sensitive behavior contrasts sharply with the robust, highly efficient (38%) RTP enabled by the head-to-tail packing of Br8-J. As illustrated by Figure 2a–d, the temperature-dependent total (i.e., prompt and delayed) PL spectra and distinct PL lifetime decay profiles of both polymorphs reveal their individual radiative and nonradiative processes. As temperature is lowered the total PL intensity and prompt PL lifetime decays of both polymorphs display opposite behavior. The PL emission from Br8-J is suppressed at low temperatures and an anomalous temperature dependence is observed in the PL lifetime decay data (Figure 2a,c). The prompt PL lifetime decay curves of the microcrystals required biexponential fitting, which is highly characteristic of the heterogeneous emission environments inherent to solid-state organics. In this model, the short-lived component (τ_2_) is attributed to excitons undergoing rapid nonradiative quenching near surface boundaries or local trap sites, while the longer-lived component (τ_1_) represents the intrinsic radiative decay of relaxed excitons within the highly ordered crystalline bulk. The amplitude-weighted average lifetime (τ_avg_) is utilized as a unified metric to track temperature-dependent changes across these competing pathways (Table S1, S2). At 77 K, the prompt τ_avg_ PL lifetime of 2.76 ns decreases slightly as the temperature increases to 275 K and abruptly increases to 4.07 ns at 295 K (Figure S3 and Table S1). A blue shift of the PL emission spectrum accompanies the abrupt increase in lifetime, suggesting a thermally activated component (Figure 2c). The increase in prompt lifetime at room temperature further indicates a more favorable environment for radiative recombination, which is crucial for achieving the higher PLQY observed at room temperature for Br8-J. In contrast, the total PL intensity of Br8-H dramatically increases at low temperature and below 175 K new vibronic structure is apparent in the PL spectra (Figure 2b). This is consistent with freezing of nonradiative vibrational pathways, enabling brighter emission that is likely more localized to one hybridized molecular orbital. The low PL intensity of Br8-H at 295 K is consistent with the low PLQY observed in the room-temperature measurements (Figure 1), and a greater prevalence of nonradiative pathways at room temperature, potentially linked to molecular packing that facilitates exciton quenching via charge transfer, for example. This is supported by the overall longer prompt PL lifetime τ_avg_ values of Br8-H compared to Br-J, attributed to slower singlet exciton recombination.

**Figure 2:**
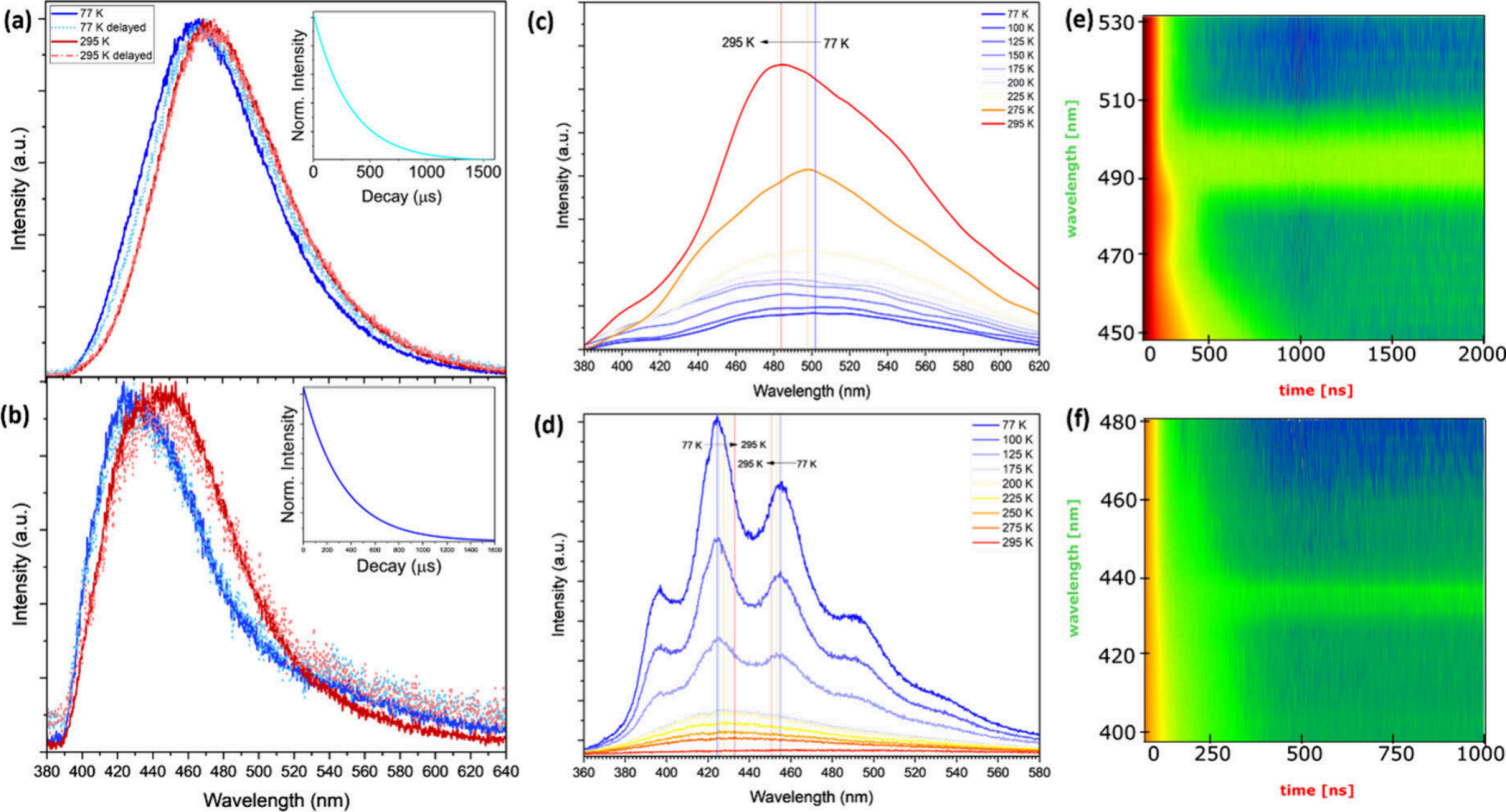
Prompt and delayed PL dynamics, temperature dependence, and time-resolved spectral mapping of single-crystal Br8 polymorphs. (a and b) Prompt (solid lines) and delayed (dashed lines) PL spectra of Br8-J (top) and Br8-H (bottom), respectively. Insets: Corresponding delayed emission decay curves highlighting the extracted delayed lifetimes. (c and d) Temperature-dependent PL behavior of the J (top) and H (bottom) polymorphs from 77 to 295 K, illustrating the evolution of emission intensity and TADF characteristics. (e and f) Two-dimensional time-resolved emission maps (lifetime on the *x* axis and emission wavelength on the *y* axis) for Br8-J and Br8-H, respectively, revealing the spectral distribution of delayed fluorescence and its temporal evolution.

Interestingly, both the Br8-J and Br8-H microcrystal dispersions also show delayed emission with lifetimes of ∼325 μs at room temperature (insets of Figure 2a,b). This observation confirms that both polymorphic forms support dual emissive pathways, prompt fluorescence and delayed phosphorescence, despite their distinct packing arrangements. Furthermore, the robust 38% quantum yield observed in Br8-J provides compelling evidence for crystal-structure-induced activation of delayed emission. Consistent with recent literature, the specific head-to-tail intermolecular interactions and rigid lattice packing in the Br8-J architecture effectively “switch on” RTP by heavily suppressing nonradiative decay pathways that otherwise dominate in the solvated or face-to-face states.^30^ Measurements were carried out on spin-coated films and microcrystals of the delayed (gated) PL spectra from both polymorphs at 295 and 77 K (Figures 2a,b, S2, and S4). It is important to note that the steady-state emission profile at 77K (Figure 2d) represents the total time-integrated emission, effectively combining both the prompt fluorescence and the distinct delayed emission components isolated in Figure 2b. Parts e and f of Figure 2 are the maps plot the emission wavelength against the luminescence lifetime to reveal the spectral distribution and temporal evolution of the delayed fluorescence. For both polymorphs, delayed emission is detected and has almost no shift in wavelength compared to the total emission spectrum. This spectral coincidence indicates that the prompt fluorescence and delayed phosphorescence originate from energetically similar excited states. In standard monomeric systems, the exchange of energy typically forces a large spectral separation between fluorescence (S_1_ → S_0_) and phosphorescence (T_1_ → S_0_). However, some theoretical models of organic solids attribute the absence of this shift to triplet excimer formation where the excited state wave function is stabilized through intermolecular orbital hybridization between adjacent molecules.^31^

Br8-J exhibits bright and persistent delayed emission at room temperature, while the delayed emission of Br8-H is weak at room temperature and increases in intensity at low temperature. This temperature-dependent behavior also suggests that the delayed emission originates from a triplet state with a slow radiative decay, characteristic of RTP. However, the generation of triplet excitons could sometimes be insufficient if they are immediately quenched by thermal vibrations.^32^ We believe that the presence of the heavy bromine atoms can facilitate ISC from singlet to triplet states, to populate the hybrid triplet state, while the rigid molecular packing restricts intramolecular motions (rotations and vibrations). This structural rigidification minimizes nonradiative energy dissipation, allowing the long-lived triplet excitons to decay radiatively.^33^

Crystallographic analysis of the reported polymorphs was conducted to gain insights into the molecular arrangement and to understand the structural features contributing to the observed emission properties. Single crystals of both polymorphs were grown using saturated solutions of Br8 in 2-methoxyethanol (Figure 3a–d) and subjected to single-crystal X-ray diffraction (XRD). Br8-J crystals grown in this way had approximate dimensions of 1 mm in length and 100 μm in diameter, whereas Br8-H crystals had approximate dimensions of 400 μm in length and 20 μm in diameter. The triclinic (*P*1̅) crystal structure of Br8-J, characterized by cell parameters *a* = 5.36155 Å, *b* = 12.7210 Å, *c* = 18.3652 Å, α = 96.819°, β = 96.808°, and γ = 92.280°, exhibits a different molecular packing compared to Br8-H, which presents a monoclinic (*P*2_1_/*c*) crystal structure with cell parameters *a* = 4.6900 Å, *b* = 33.3655 Å, *c* = 7.9128 Å, α = 90.0° β = 98.145°, and γ = 90.0°. A comparison of the crystal 2D diffraction patterns is presented in Figure 3e. The Br8-H dimer exhibits a centroid-to-centroid distance of 4.69 Å and a slip angle of 86.57°, placing it firmly in the H-aggregate regime (θ > 54.7°); see Figure S11 and Table S6. In contrast, the head-to-tail translation in Br8-J of 5.35 Å (centroid to centroid) and a slip angle of 44.33°, structurally defining a J-aggregate (θ < 54.7°). While classical J-aggregates exhibit near-zero Stokes shifts, the large Stokes shifts observed in the Br8 system are attributed to heavy-atom-induced ISC and subsequent structural relaxation, resulting in emission from lower-energy triplet or excimer-like states rather than the initial rigid singlet exciton. The notable differences in unit cell parameters and crystal symmetry between Br8-J and Br8-H indicate distinct molecular packing arrangements in their respective crystalline forms. The triclinic P-1 symmetry of Br8-J suggests a more elongated and needle-like molecular arrangement, possibly facilitating efficient charge transport and emission characteristics observed in its PL spectra. In contrast, Br8-H exhibits a monoclinic symmetry with altered cell dimensions, reflecting a twisted and potentially more compact molecular packing that correlates with its different optical properties, such as higher bandgap and reduced PL intensity. The packing-dependent optical divergence observed in the Br8 system closely parallels established cases of organic color polymorphism, such as the classic conformational polymorphs of dimethyl 2,5-dichloro-3,6-dihydroxyterephthalate.^34^ Much like how that reference system relies on drastic shifts in dihedral angles and halogen-mediated interactions (Cl···H) to dictate its distinct planar-yellow and twisted-white architectures, Br8 leverages structural isomerization (*all-trans* versus *single-gauche*) alongside specific Br···H contacts to lock into its respective triclinic (Br8-J) and monoclinic (Br8-H) lattices. Ultimately, these packing-driven conformational variations strictly dictate the intermolecular orbital overlap, serving as the structural toggle between the deep-blue fluorescence of the H-aggregate and the highly efficient, cyan RTP of the J-aggregate. To summarize the geometry-driven photophysics, the distinct electronic pathways of the two polymorphs are mapped out in the proposed Jablonski diagrams (Figure S12). According to Spano,^35^ it is possible to observe strong spatial coupling between Frenkel excitons and intermolecular CT species depending on the precise molecular slip angle. This provides a rigorous theoretical basis for why only the specific head-to-tail geometry of Br8-J allows for the stabilization of the long-lived, excimer-like triplet states responsible for our observed highly efficient RTP. Conversely, the face-to-face stacking of the Br8-H aggregate dictates an excitonic coupling regime that leaves the system highly susceptible to the dominant nonradiative thermal quenching pathways observed at ambient temperatures.

**Figure 3:**
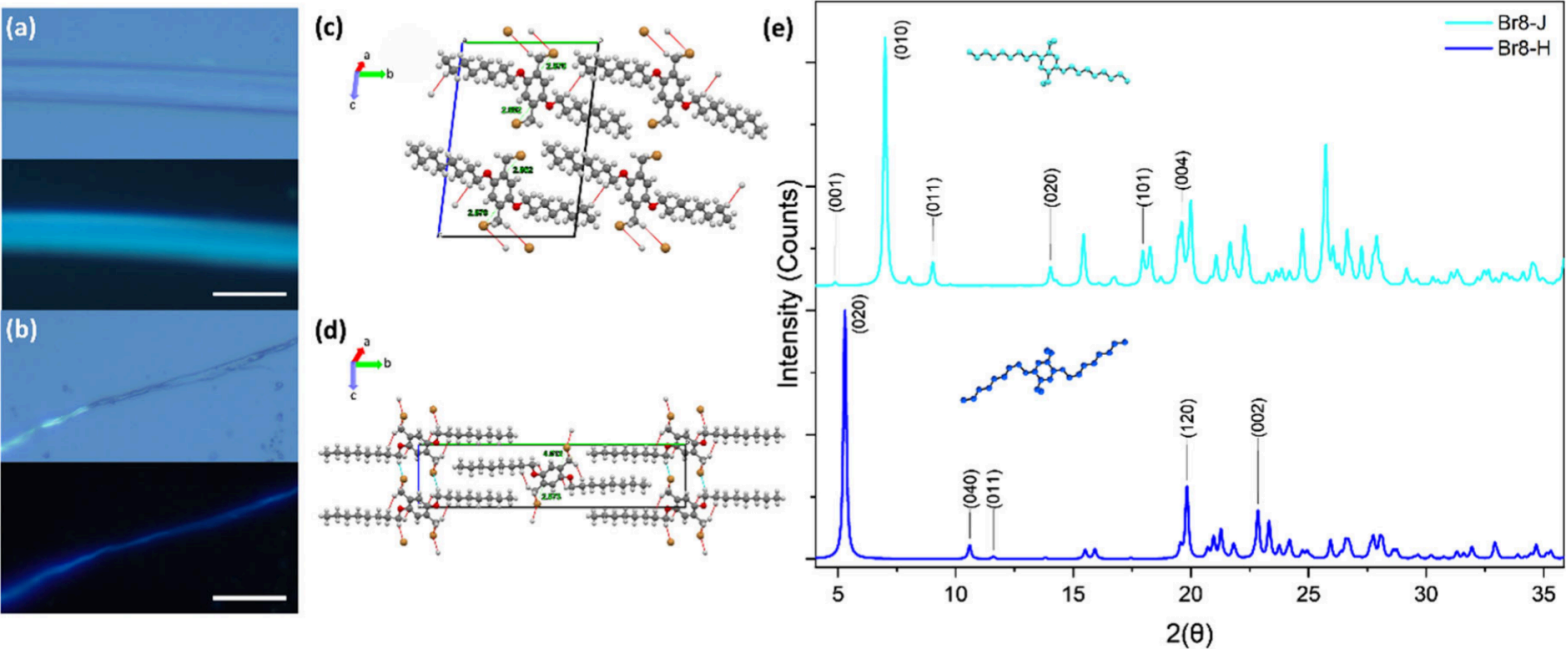
Single-crystal XRD analysis of Br8-H and Br8-J. (a and b) Optical microscopy of single crystals: bright-field (top) and fluorescence (bottom) imagesof (a) Br-J and (b) Br8-H (the scale bars are 100 μm). (c) Br8-J and (d) Br8-H crystal structure determined from single-crystal analysis showing the three-dimensional representation of the unit cells illustrating the spatial arrangement of molecules within the crystal lattice. (e) Observed XRD patterns of Br8-J (cyan) and Br8-H (blue) crystals, showing distinct diffraction peaks corresponding to their respective crystal structures. The inset shows the molecular structure of Br8 in *all-trans* and *single-gauche* conformations, as observed in polymorphs J and H, respectively.

Investigation of the Br8 single crystals using XRD revealed highly ordered molecular arrangements, which exhibited both intra- and intermolecular interactions, including an intermolecular Br···H interaction of 3.01 Å for Br8-J. This specific halogen interaction likely plays a crucial dual role in the Br8 crystals: contributing to the overall molecular packing and solid-state (suppressing nonradiative vibrational decay), and it physically forces the nonbonding (n) orbitals of the heavy bromine atoms into close proximity with the adjacent aromatic π systems. The emission features of these crystals are strongly governed by this ordered molecular arrangement, and the RTP properties could be attributed to a restrained nonradiative relaxation through close nearest-neighbor intermolecular interactions. Time-dependent density functional theory (TD-DFT) simulations further support this by showing the presence of a T_2_ state lower in energy than the S_1_ state, which may undergo rapid internal conversion to T_1_, helping to populate the long-lived triplet state responsible for the observed RTP. Figure 3c, d show the crystal packing in the plane of the aromatic rings. The close proximity of molecules in the highly ordered Br8 crystal, facilitated by both the halogen and hydrogen interactions, also raises the possibility of through-space charge transfer,^36^ which occurs when there is a direct overlap of electron orbitals between neighboring molecules that are not covalently bonded. Using the nearest aromatic carbons for a geometrical comparison, both Br8-J and Br8-H exhibit intermolecular aromatic ring distances that are not exactly identical (4.81 and 4.69 Å, respectively). Br8-J exhibits significantly more nonbonding nearest neighbor interactions compared to Br8-H (Figures S5 and S6 and Tables S3 and S4). The packing differences between Br8-J and Br8-H significantly impact their photophysical properties, particularly with respect to RTP and luminescence behavior. In Br8-J, the stronger ISC facilitated by the bromine interactions and orbital hybridization supports a more pronounced RTP character, enabling more efficient phosphorescence at room temperature compared to Br8-H. This is likely due to the favorable packing arrangement that promotes effective SOC and excited-state stabilization.^37^

In contrast, Br8-H only exhibits relatively efficient luminescence below 175 K, suggesting that its packing arrangement does not support efficient RTP at higher temperatures. The comparatively less favorable packing in Br8-H likely prevents the necessary interactions for ISC, limiting its luminescent behavior to lower temperatures where thermal activation and vibrations are reduced, and phosphorescence is less hindered by nonradiative recombination. Therefore, the packing in Br8-J is more conducive to RTP, while Br8-H is more temperature-sensitive and only shows efficient long-lived luminescence under colder conditions.

To theoretically evaluate this mechanism, TD-DFT calculations employing the Tamm–Dancoff approximation (TDA-DFT)^38,39^ were performed to study the electronic energy levels in Br8 (see Methods and Figures 4, S7, and S8). TDA-DFT was selected given its reliability predicting singlet–triplet gaps.^40^ We considered dimers (i.e., nearest-neighbor molecular pair) obtained from Br8-J and Br8-H crystal structures. It is important to note the inherent limitations of this approach: these dimer-level calculations do not capture the extended, many-molecule excitonic coupling or the full band structure of macroscopic J- and H-aggregates. Rather, they serve as a qualitative illustration of the local orbital overlap occurring at the primary interaction nodes (Figures S7 and S8).

**Figure 4:**
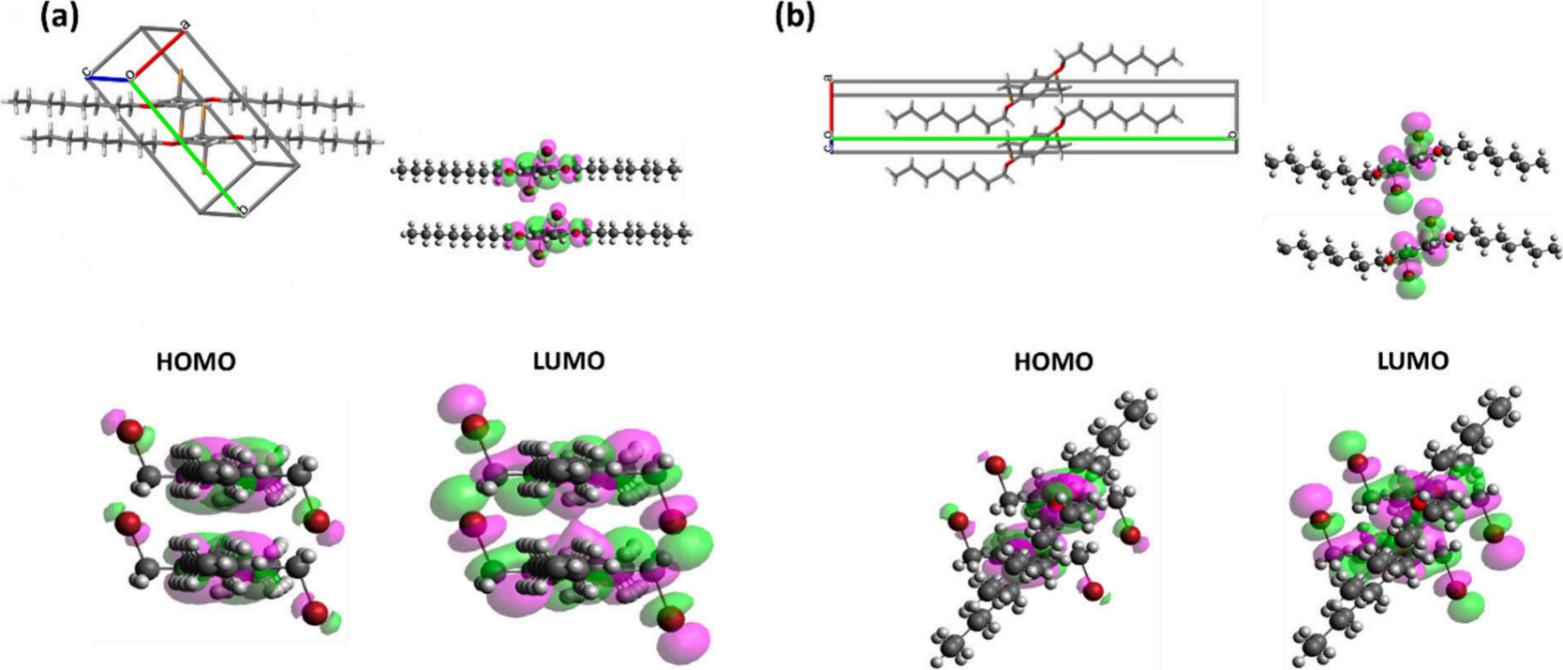
TDA-DFT analysis of (a) Br8-J and (b) Br8-H systems. Top: Representative dimer packing motifs extracted from the crystal lattice and their corresponding calculated dimer molecular orbitals. Bottom: Canonical HOMO and LUMO frontier molecular orbitals.

Figure 4 shows the TDA-DFT results for both Br8-J and Br8-H dimers. Both systems present a near zero singlet–triplet energy gap when considering the lowest singlet states and the higher-lying T_3_ and T_4_ triplet states. Canonical frontier orbital analysis reveals significant electron density localized on the bromine atoms, particularly within the lowest unoccupied molecular orbitals (LUMOs). The simulations confirm the presence of intermolecular hybridized orbitals within the chosen dimer pairs, reflecting an increased mixing between the π* orbitals of the aromatic ring and the bromine lone pairs, possibly contributing an n → π* character to the transitions. This hybridization provides the theoretical basis for the observed photophysics: the spatial delocalization of the hybridized exciton minimizes the electron–hole exchange energy, significantly reducing the singlet-triplet energy splitting. Furthermore, the bromine atoms can induce strong SOC,^41,42^ facilitating efficient ISC. Combined with the small singlet–triplet energy gaps observed in both systems and low energy T_1_ states, these properties suggest that both compounds can function as RTP emitters. This bromine interaction enhances SOC, leading to stronger ISC between singlets and triplets. We also observe increased mixing between the π* orbitals of the aromatic ring and the bromine lone pairs, Ultimately, the oberserved intermolecular orbital overlap provides a theoretical basis for the formation of stabilized, excimer-like triplet states, aligning with our experimental observation of spectrally congruent prompt and delayed emission profiles.

## Conclusions

The solid-state structures and photophysics of two crystalline polymorphs of the halide-containing molecule Br8 were investigated: one with deep-blue emission 438 nm (Br8-H) and the other with lower-energy 467 nm emission (Br8-J). The two polymorphs were obtained by changing the cooling rate of heated solutions. While the Br8-J polymorph achieved a remarkable quantum yield of 38% in the microcrystalline state, the face-to-face packing of the Br8-H polymorph resulted in a significantly lower quantum yield of 2% for the microcrystals and 0.2% for the thin films, highlighting the critical role of intermolecular interactions in suppressing nonradiative recombination. The PL lifetime of both Br8-J and Br8-H showed both short (∼4 ns) and long (∼325 μs) components that occur at approximately the same wavelengths corresponding to both fluorescence and RTP, respectively. The emission of Br8-J blue-shifted slightly by 7 nm when cooled to 77 K indicating a small thermally activated component of the emission. The Br atoms within the molecular structure exhibited different distances from the conjugated organic unit and the intermolecular spacing varied for each polymorph. In Br8-J, the intermolecular Br···H distance was 3.01 Å providing stronger intermolecular interactions compared to Br8-H. Additionally, the *all-trans* chain arrangement in Br8-J results in a less twisted, lower energy molecular conformation compared to Br8-H. This structure is consistent with lower energy emission and the higher quantum yield of Br8-J due to stronger intermolecular interactions that suppress vibrational nonradiative recombination. For Br8-H, each Br atom is ∼3.4 Å from a hydrogen on the neighboring molecule indicating weaker intermolecular interactions and potentially lower quantum yield. Br8-H exhibits a gauche arrangement for one torsional angle near the chain attachment site, leading to a twisted, higher-energy molecular conformation. This is consistent with the higher energy emission. The observed prompt decay elongation and efficient delayed emission observed in Br8-J crystals implies that heavy atom effects and SOC play a significant role in Br8-J emission, promoting ISC and enabling RTP behavior, whereas Br8-H lacks these features. In our system RTP and prompt emission are very close in energy to each other; therefore, significant hybridization and energy exchange is likely between triplet and singlet states. In contrast, Br8-H demonstrates limited luminescence, which is confined to low temperatures, likely due to less efficient packing and a lack of the favorable intermolecular interactions present in Br8-J. These findings highlight the importance of crystal packing, molecular conformation, and orbital interactions in governing the temperature dependence of luminescent behavior of halide-containing organic crystals, providing insights for the design of organic materials with tunable RTP.

## Methods

To initiate the first crystalline form of 1,4-bis­(bromomethyl)-2,5-bis­(octyloxy)­benzene (Br8), Br8-J, 4 mg of Br8 (Sigma-Aldrich, ≥99.0%, molecular weight of 520.4 Da) was dissolved in 1 mL of 2-methoxyethanol in an amber glass vial (see Table S5 and Figure S9 for the solvents investigated). The solution underwent alternating cycles of sonication (5 min) and heating in a water bath at 85 °C (5 min) until it became visibly clear with no aggregates. Then, the solution was allowed to cool to room temperature at a relatively slow cooling rate (∼10 °C/h) in order to promote formation of needle-like crystals. To fabricate the second crystalline form of Br8, Br8-H, 4 mg of Br8 was dissolved in 1 mL of 2-methoxyethanol in an amber glass vial. The solution underwent three alternating cycles of sonication (5 min) and heating in a water bath at 85 °C (5 min) while stirring at 500 rpm, until it became clear. The total time for this process was 30 min. Then, the solution was rapidly cooled to 7 °C leading to the formation of thread-like crystals. For absorption and PL measurements, the same process for formation of Br8-J and Br8-H solutions was employed, but with a starting concentration of 4 mg/mL. Subsequently, the needle-like and thread-like crystal solutions were diluted to a concentration of 0.01 mg/mL for PLQY measurements. The samples for optical imaging were prepared by drop-casting the 4 mg/mL microcrystal dispersions onto clean glass microscope slides and allowing the 2-methoxyethanol solvent to completely evaporate under ambient conditions prior to examination (Figure S10), while the samples for absorption and PL spectroscopy were from 0.01 mg/mL crystal solutions. Recrystallization of the XRD single crystals was achieved by dissolving 15 mg of Br8 in 1 mL of 2-methoxyethanol, followed with heating and cooling, as mentioned above (Figure S9).

To develop thin films, we employed a two-solvent approach starting with a tetrahydrofuran (THF) solution of 20 mg/mL of Br8, followed by the addition of a secondary solvent, 2-methoxyethanol, to promote crystallization. The conditions for spin-coating involved a two-step process: an initial deposition at 2000 rpm (15 s) to deposit the THF solution, followed by a secondary deposition at 4000 rpm (30 s) to apply 2-methoxyethanol. This method ensured the formation of high-quality, luminescent thin films suitable for photophysical analyses and optoelectronic applications.

Absolute PLQYs were determined using a spectrometer (Horiba Fluorolog) equipped with an integrating sphere to account for the varying scattering properties of the solid-state samples. The reported PLQY values carry an estimated instrumental error of 0.3–1.8% (Table S7).

Temperature-dependent PL spectra and TRPL decay profiles were also recorded on crystals and 80 nm thin-film samples on quartz with a time-correlated single-photon-counting instrument using the 340 nm pulsed light from a Pharos/Orpheus Light Conversion laser system and a time analyzer (TimeHarp 260 nano, Picoquant Germany). PL decays were individually fit model with the Fluofit Picoquant software using a biexponential fit model with lifetime contributions calculated as averaged intensities.

The crystal structures for the two compounds were investigated by single-crystal diffraction. Small fragments (edge < 50 μm) from the syntheses of Br8-H (2448223) and Br8-J (2448236) were examined using finely collimated Cu Kα radiation (λ = 1.54184 Å) at room temperature with a Rigaku XtaLAB Synergy-S diffractometer system. Reflection data to 0.78 Å resolution (θ = 80°) using Cu Ka (1.5418 Å) X-radiation were processed with the CrysAlisPro software. The structure solution program SHELXT, least-squares program SHELXL, and GUI program ShelXle were used for structure determination and refinement. The powder XRD patterns were collected in the 2θ range between 15° and 120° by an X’Pert Philips diffractometer for Br8-J and a Bruker D8 Advance diffractometer for Br8-H, both employing Cu Kα radiation (λ = 1.54184 Å) and a step size of 0.02° (2θ). The structural refinements by the Rietveld method were performed using the FullProf software suite. The CCDC Mercury program was used to visualize the structural models and create all crystallographic figures. Calculated precession photographs of the 0*kl*, *h*0*l*, and *hk*0 zones of reflection data collected for Br8-H and Br8-J up to 0.5 Å resolution are given in Figure S10a,b.

TD-DFT calculations employing TDA-DFT^38^ were performed at the B3LYP-D4/def2-TZVP level of theory,^43–45^ as implemented in ORCA 5.0.4.^39^ TDA-DFT was selected given its reliability predicting singlet–triplet gaps.^41^

## Supplementary Material



## Notes

Time-resolved PL decay curves and exponential fitting parameters, temperature-dependent excitation spectra, crystallographic packing parameters, and supplementary structural and optical characterization data (PDF)
