# Supplementary material for: Polymorph-Dependent Photophysics of Blue-Emitting Brominated Organic Crystals
Source: ACS Appl Opt Mater. 2026 Jun 15;4(6):1689–98. doi: 10.1021/acsaom.6c00105 (PMC13316979; doi:10.1021/acsaom.6c00105)
Supplement: Supplementary file 1 [file ot6c00105_si_001.pdf]

Supporting Information

Polymorph-Dependent Photophysics of Blue-Emitting Brominated Organic Crystals

Pacheco, Haydee [1], Valdiviezo, Jesus [2,3,4], De Leon, Rianne G. [1], Emge, Thomas J. [6], Cotlet, Mircea [5], O'Carroll, Deirdre M. [1,6*]

[1] *Department of Materials Science and Engineering, Rutgers University, 607 Taylor Rd., Piscataway, NJ 08854, USA*

[2] *Department of Biological Chemistry and Molecular Pharmacology, Harvard Medical School, Boston, Massachusetts 02115, United States;*

[3] *Department of Cancer Biology, Dana-Farber Cancer Institute, Boston, Massachusetts 02215, United States*

[4] *Sección Química, Departamento de Ciencias, Pontificia Universidad Católica del Perú, San Miguel, Lima 15088, Peru*

[5] *Center for Functional Nanomaterials, Brookhaven National Laboratory, Upton, NY 11973, USA*

[6] *Department of Chemistry and Chemical Biology, Rutgers University, 123 Bevier Rd., Piscataway, NJ 08854, USA*

**Corresponding author: ocarroll@rutgers.edu*

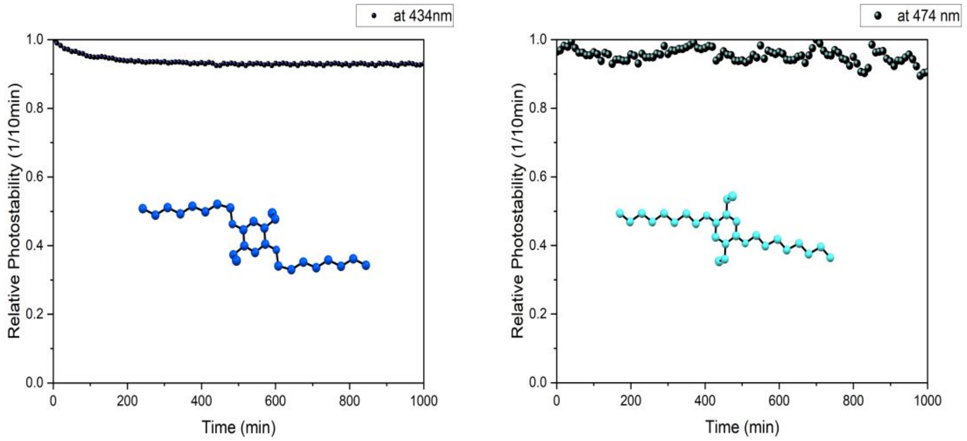

Figure S1. Photostability of Br8-H (left) and Br8-J (right) thin films for over 16 h (excitation at 365 nm).

We tested the photostability of Br8 polymorph films under continuous illumination at a wavelength of 365 nm and an intensity of 6 mW/cm^2. Both encapsulated and non-encapsulated samples were studied to assess the role of environmental protection on material performance. Initial results indicate that the Br8 polymorphs exhibit excellent photostability, retaining over 80% of their photoluminescence intensity after 16 hours of exposure. The encapsulated samples showed minimal photodegradation. In contrast, the non-encapsulated films experienced a significant decline in luminescence, primarily attributed to surface photo-oxidation.

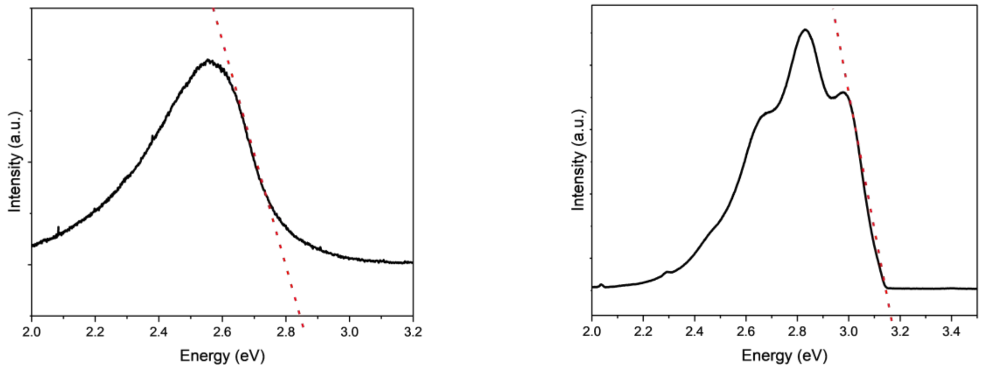

Figure S2. Estimation of the optical energy gaps from solid-state photoluminescence. High-energy emission onset approximations for the (left) Br8-J and (right) Br8-H polymorphs.

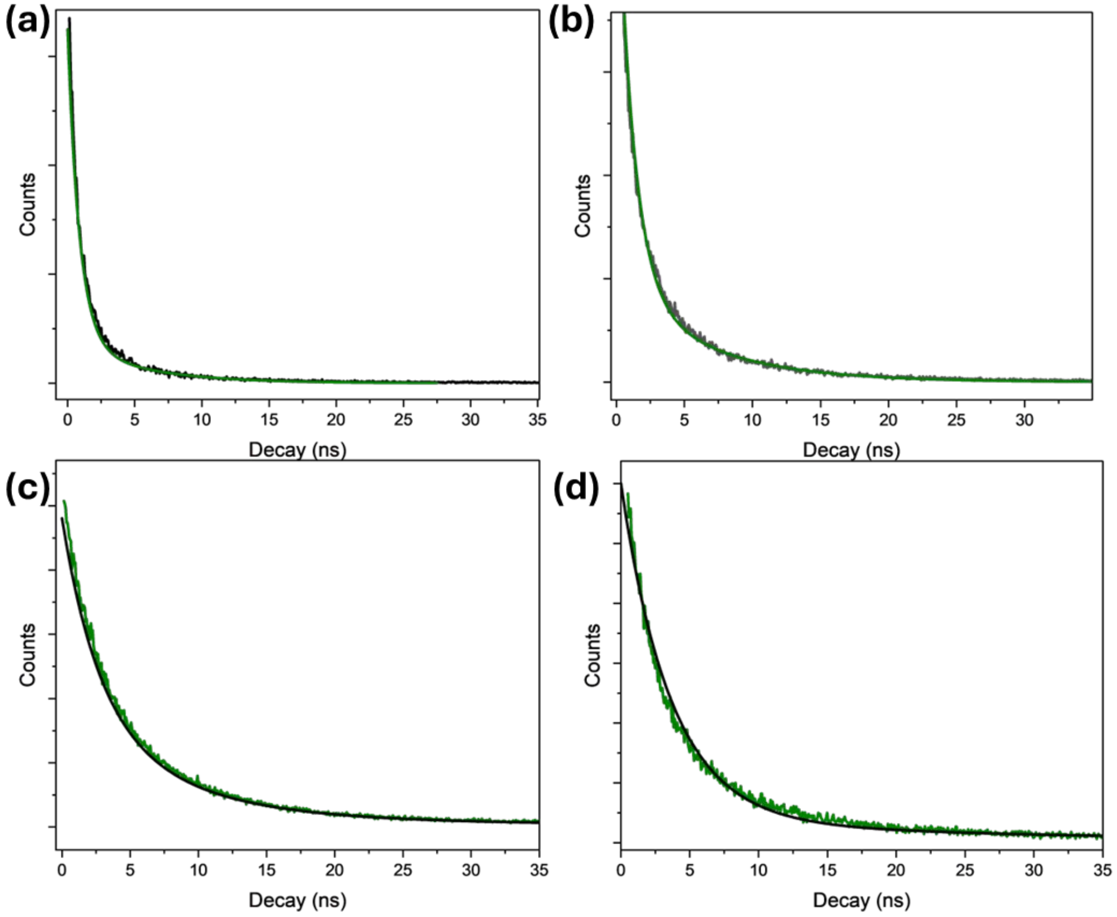

Figure S3. Representative time-correlated single photon counting (TCSPC) nanosecond-range (i.e. prompt) photoluminescence decay curves for Br8-J and Br8-H microcrystals at room temperature. (a) Br8-J at 77 K, (b) Br8-J at 295 K, (c) Br8-H at 77 K, and (d) Br8-H at 295 K. The green solid lines represent the bi-exponential fits used to extract the prompt lifetimes. Detailed fitting parameters, goodness-of-fit (χ^2) values, and calculated error margins are provided in Tables S1 and Table S2.

Table S1. The prompt intensity-weighted PL lifetime (average lifetime, percentage error of the average lifetime, lifetime components (τ_1, τ_2) intensity percentages of each lifetime component (I_1, I_2), goodness-of-fit (χ^2) of Br8-J as a function of temperature. Values in parenthesis represent the standard deviation.

Temp. (K)	**Average Lifetime (ns)**	**Error (%)**	**τ_1 (ns)**	**τ_2 (ns)**	**I_1 (%)**	**I_2 (%)**	**χ^2**
77	2.76	9.4	5.31 (±0.17)	0.87 (±0.03)	42.65 (±0.91)	57.35 (±1.30)	0.75 (±0.22)
100	2.60	8.6	4.98 (±0.15)	0.84 (±0.028)	42.69 (±1.38)	57.31 (±0.95)	0.92 (±0.25)
125	2.66	10.5	4.72 (±0.15)	0.77 (±0.032)	47.65 (±0.96)	52.35 (±0.21)	0.70 (±0.22)
150	2.86	9.1	5.06 (±0.15)	0.78 (±0.030)	48.63 (±2.60)	51.37 (±1.70)	0.60 (±0.20)
175	2.89	31.7	5.08 (±0.03)	0.79 (±0.196)	48.95 (±1.64)	51.05 (±0.93)	0.56 (±0.27)
200	2.65	8.0	4.79 (±0.14)	0.75 (±0.028)	47.06 (±1.88)	52.94 (±1.36)	0.68 (±0.23)
225	2.59	9.0	4.55 (±0.14)	0.70 (±0.029)	49.01 (±1.50)	50.99 (±2.82)	0.57 (±0.19)
250	2.76	8.6	4.71 (±0.15)	0.71 (±0.032)	51.24 (±1.50)	48.76 (±0.94)	0.57 (±0.20)
275	2.99	8.2	4.80 (±0.15)	0.71 (±0.034)	55.69 (±2.78)	44.31 (±1.88)	0.54 (±0.19)
295	4.07	14.8	6.48 (±0.13)	1.18 (±0.040)	54.41 (±3.54)	45.59 (±2.20)	1.17 (±0.38)

Table S2. The prompt intensity-weighted PL lifetime (average lifetime, percentage error of the average lifetime, lifetime components (τ_1, τ_2) intensity percentages of each lifetime component (I_1, I_2), goodness-of-fit (χ^2) of Br8-H as a function of temperature. Values in parenthesis represent the standard deviation.

Temp. (K)	**Average Lifetime (ns)**	**Error (%)**	**τ_1 (ns)**	**τ_2 (ns)**	**I_1 (%)**	**I_2 (%)**	**χ^2**
77	17.168	18	2.68 (0.22)	7.68 (0.32)	29.27 (6.27)	50.17	0.80 (0.33)
100	16.877	18.6	46.29 (0.17)	4.65 (2.8)	29.37 (6.5)	70.63 (3.65)	0.92 (0.33)
125	17.34	5.49	43.16 (2.03)	4.23 (0.14)	33.67 (6.06)	66.33 (7.60)	0.96 (0.33)
150	18.1	4.8	42.85 (1.93)	3.89 (0.14)	36.47 (5.77)	63.53 (3.27)	0.83 (0.28)
175	18.75	4.44	42.04 (1.83)	3.54 (0.13)	39.51 (5.75)	60.49 (4.08)	0.85 (0.29)
200	19.89	4.54	41.46 (1.78)	3.38 (0.14)	43.37 (5.47)	56.63 (5.83)	0.86 (0.29)
225	22.08	4.24	42.82 (1.79)	3.21 (0.14)	47.64 (4.17)	52.36 (3.07)	0.82 (0.28)
250	22.868	4.95	41.10 (0.15)	3.12 (1.64)	48.00 (4.73)	52.00 (4.17)	0.87 (0.31)
275	23.24	4.18	40.10 (1.55)	3.09 (0.15)	54.44 (4.18)	45.56 (4.26)	0.85 (0.30)
295	12.5	2.71	28.96 (2.33)	3.61 (0.29)	35.45 (6.28)	64.55 (4.20)	0.88 (0.25)

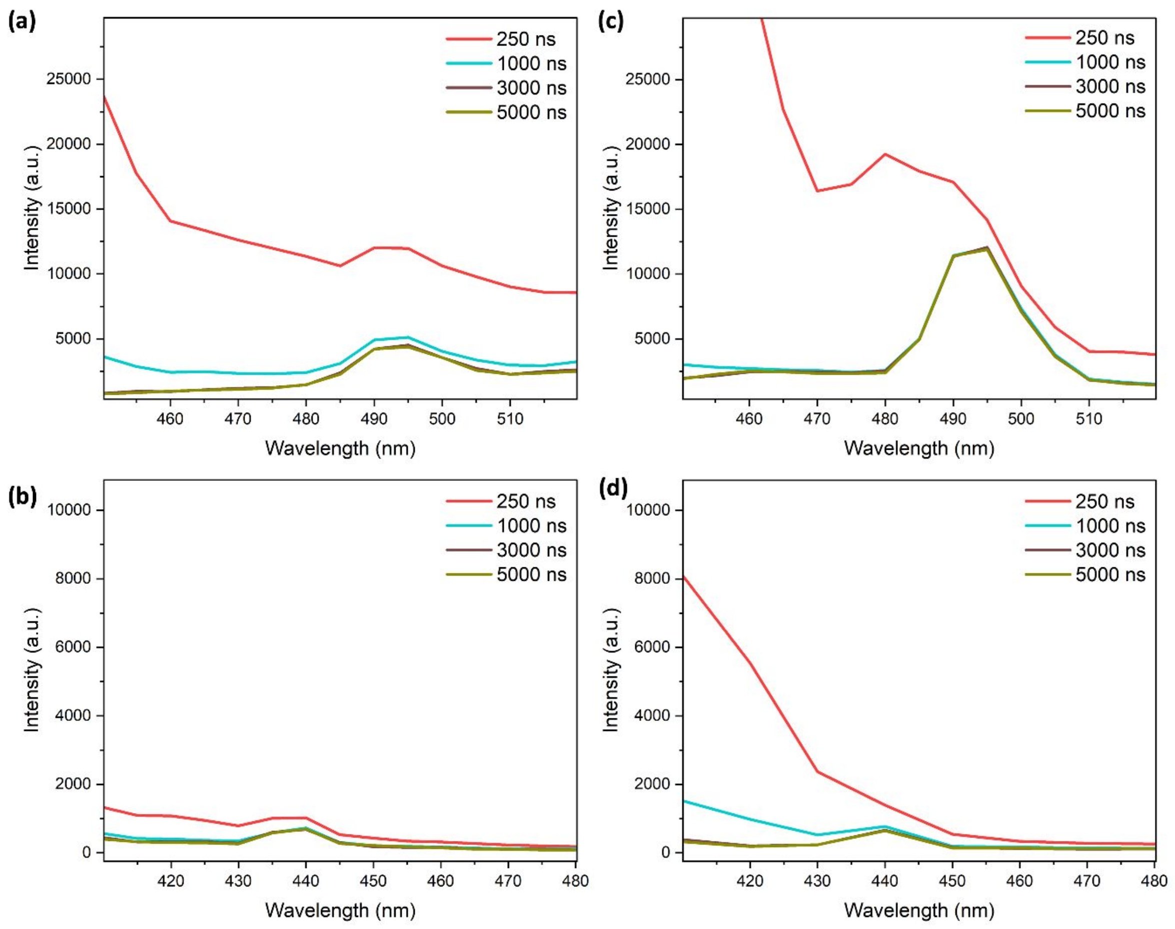

Figure S4. Time-resolved PL spectra at various time frames for (a,c) Br8-J and (b,d) Br8-H at (a,b) 77 K and (c,d) 295 K.

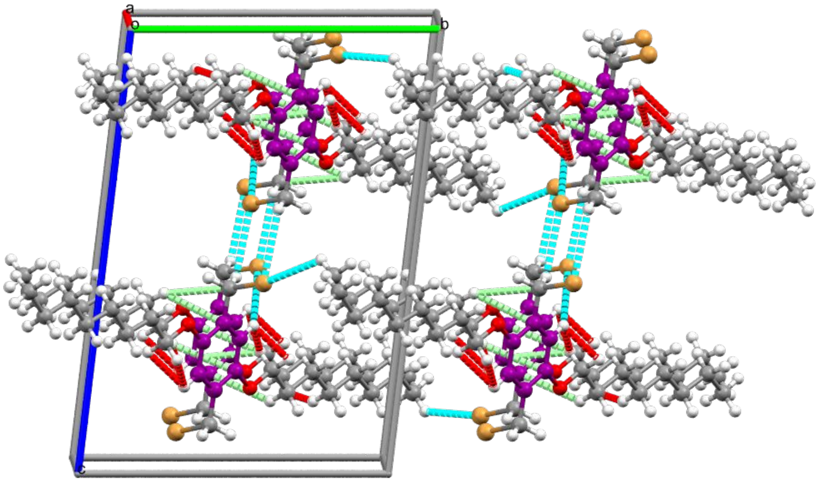

Figure S5. Nearest neighbor interatomic distances in Br8-J crystals for contacts shorter than the sum of the Van der Waals radii plus 0.1 Å (intramolecular and intermolecular), and for contacts separated by >4 bonds (intramolecular). Contacts are indicated by dashed lines: Br…H (blue), H…H (red), C…H (green) and C…C (pink). Aromatic C atoms (purple), aliphatic C atoms (grey), H atoms (white), Br atoms (yellow) and O atoms (red).

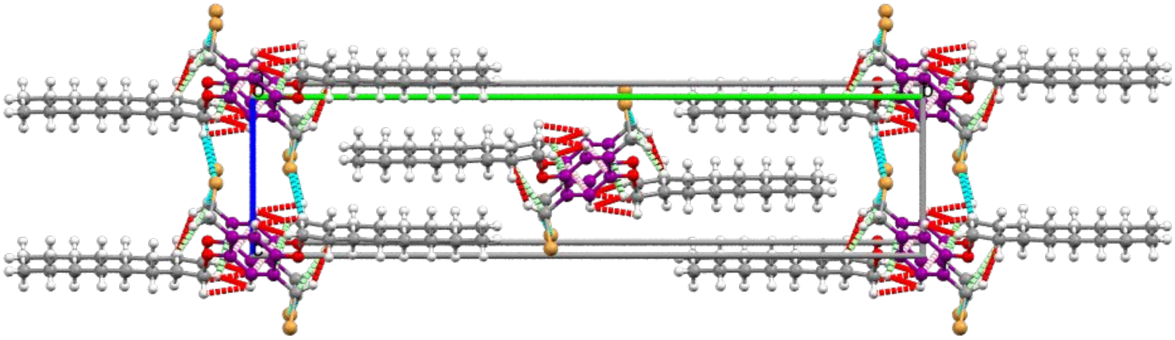

Figure S6. Nearest neighbor interatomic distances in Br8-H crystals for contacts shorter than the sum of the Van der Waals radii plus 0.1 Å (intramolecular and intermolecular), and for contacts separated by >4 bonds (intramolecular). Contacts are indicated by dashed lines: Br…H (blue), H…H (red) and C…H (green). Aromatic C atoms (purple), aliphatic C atoms (grey), H atoms (white), Br atoms (yellow) and O atoms (red).

Table S3. Nearest neighbors distances for Br8-J from XRD data.

Number	Atom1	Atom2	Length (Å)	Length-VdW (Å)
1	Br1	H16C	3.102	0.052
2	H24C	Br2	3.05	0
3	H3	Br1	3.138	0.088
4	H3	H17A	2.262	-0.138
5	H3	H17B	2.234	-0.166
6	H6	H9A	2.285	-0.115
7	H6	H9B	2.313	-0.087
8	H23B	H9A	2.392	-0.008
9	C2	H10B	2.952	0.052
10	C4	H9B	2.958	0.058
11	C7	H10B	3	0.1
12	H17A	C1	2.919	0.019
13	H17A	C6	2.984	0.084
14	H18B	C5	2.932	0.032
15	H20A	H18A	2.46	0.06
16	H22A	H22A	2.446	0.046
17	H7B	C16	2.961	0.061
18	Br2	H8A	3.01	-0.04
19	Br1	H7A	3.081	0.031

Table S4. Nearest neighbors distances for Br8-H from XRD data.

Number	Atom1	Atom2	Length	Length-
1	H005	H00A	2.373	-0.027
2	H005	H00B	2.266	-0.134
3	C003	C005	3.418	0.018
4	C004	H00B	2.963	0.063
5	H00B	C003	2.87	-0.03
6	C007	H00J	2.99	0.09
7	H00C	H00J	2.357	-0.043
8	H00D	Br01	2.936	-0.114
9	Br01	H00A	2.97	-0.08

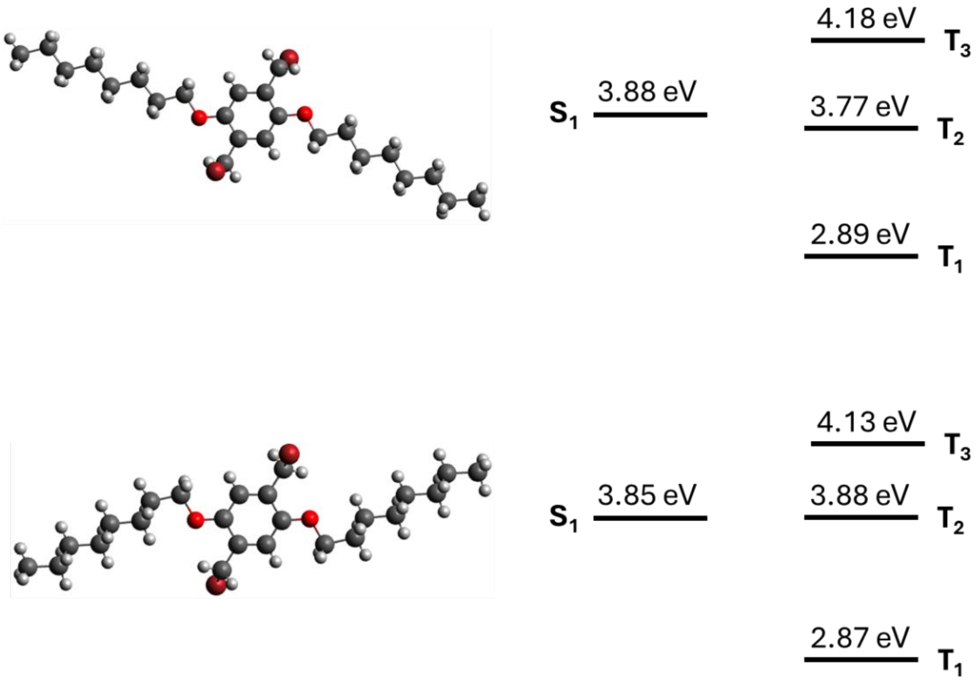

Figure S7. Theoretically calculated singlet and triplet states for the monomeric forms of Br8-J (top) and Br8-H (bottom).

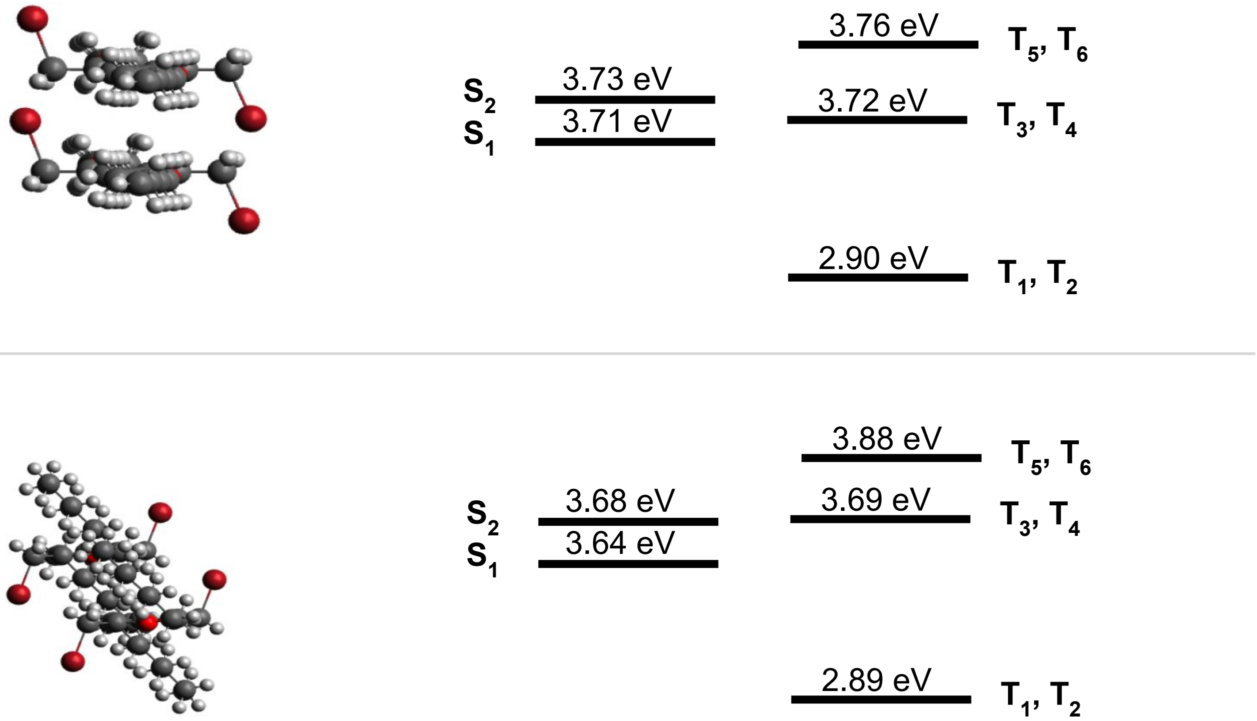

Figure S8. Theoretically calculated singlet and triplet states for the dimeric forms of Br8-J (top) and Br8-H (bottom).

Table S5. Solvents used, their Hansen Solubility Parameter values, and observed behaviors in the crystallization of (Br8) blue emitters.

Solvent	δD (MPa1/2)	δP (MPa1/2)	δH (MPa1/2)	δt (MPa1/2)	Observed Behavior (Estimated)	Solution Saturation (Estimated)
Hexane	14.9	0.0	0.0	24.1	Very poor solubility, likely precipitating solute	Low
Methanol	15.1	12.3	22.3	29.6	High solubility; crystallization	High
Ethanol	15.8	8.8	19.4	26.5	Better-ordered films, higher charge mobility	High
2-Methoxyethanol	16.2	9.2	16.4	24.8	Good solubility, uniform film formation	High
Tetrahydrofuran (THF)	16.8	5.7	8.0	19.4	Glass-like form, useful as an antisolvent	Moderate
Chloroform	17.8	3.1	5.7	19.0	Enhanced crystallization, slow evaporation	High
Toluene	18.0	1.4	2.0	18.6	Moderate solubility, uniform films	Moderate

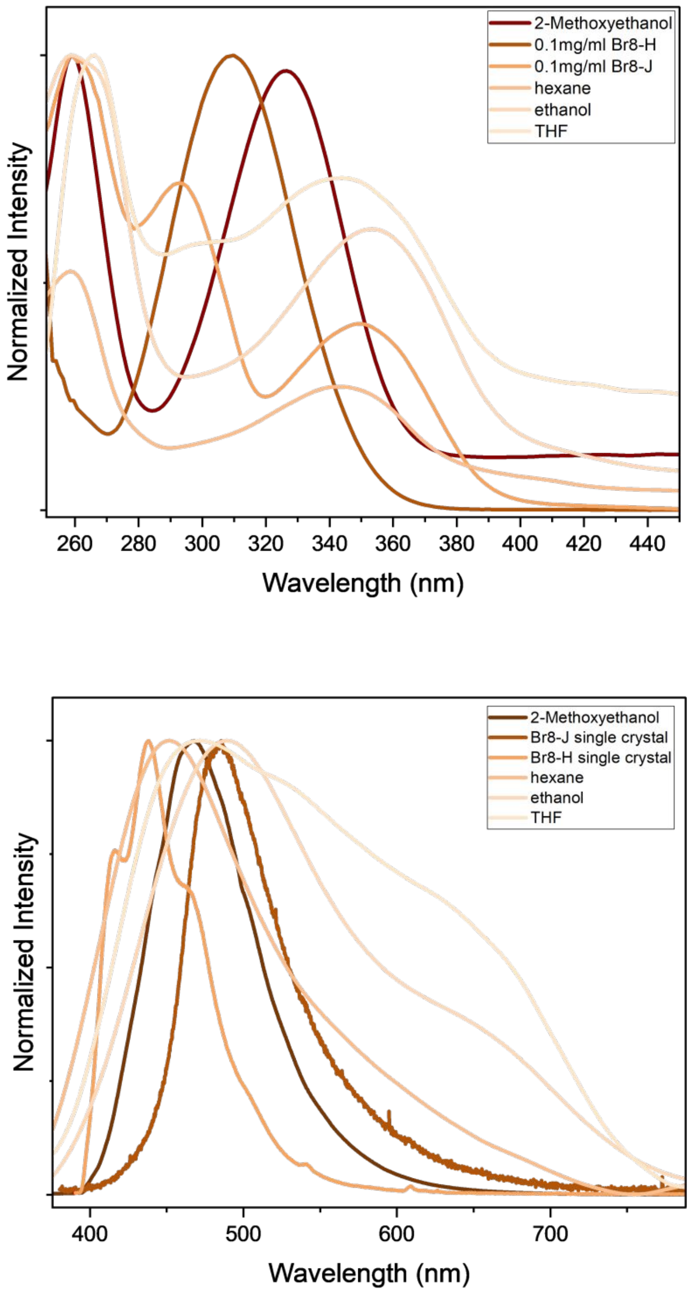

Figure S9. Solvatochromic study of the Br8 monomer. (Top) Absorption spectra of the Br8 monomer recorded in various solvents at a concentration of 10^{-5} M under ambient conditions along with the absorption of the microcrystals (0.1 mg/mL). (Bottom) Monomer

photoluminescence (PL) spectra in various solvents at a concentration of 10^{-5} M under ambient conditions along with the single crystal PL spectra.

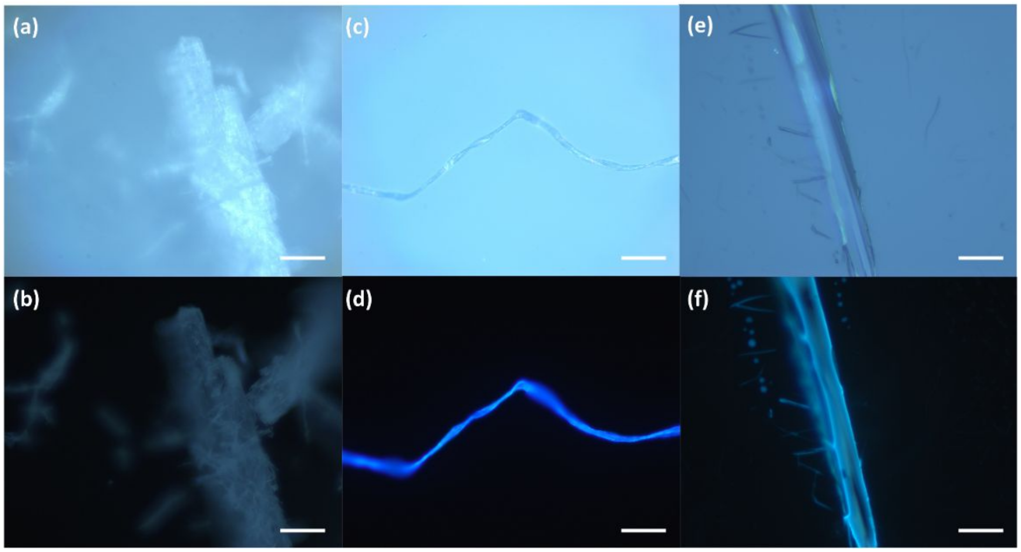

Figure S10. 1,4-dibromo-2,5-dioctyloxybenzene (Br8) powder (a,b), Br8-H crystals (c,d) and Br8-J crystals (e, f) under ambient light (top), or 365 nm excitation (bottom). Scale bar is 100 μm.

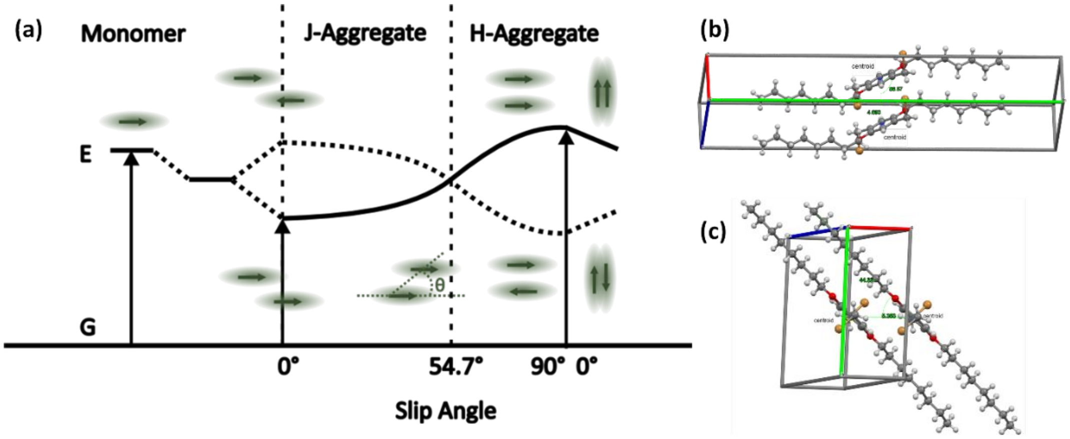

Figure S11. Structural and energetic diagram for the excitonic coupling in Br8 polymorphs. (a) Schematic energy level diagram based on Kasha's exciton model. (b, c) Crystallographic illustrations extracted from single-crystal X-ray diffraction data. The images depict the calculated center-to-center intermolecular distances (R) and the corresponding Kasha slip angles θ measured relative to the transition dipole axis.

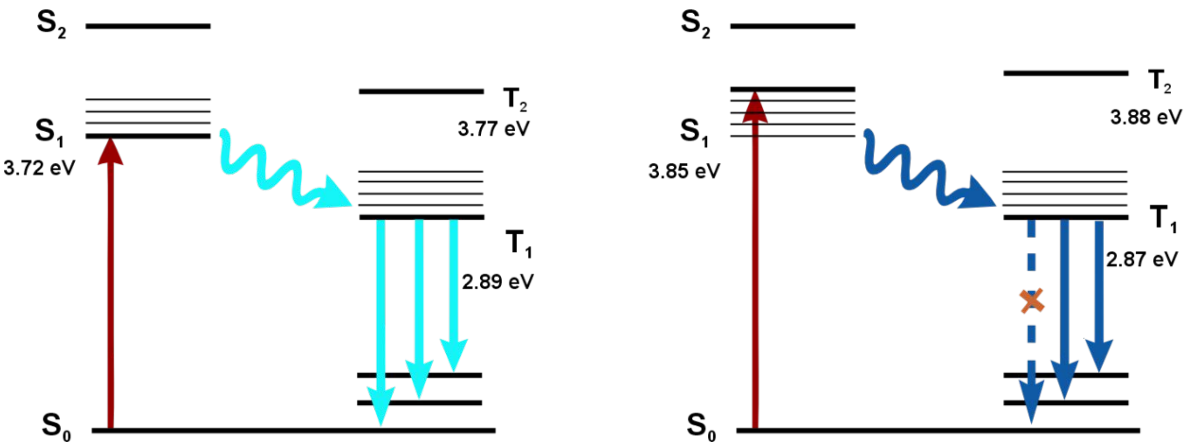

Figure S12. Energy level diagrams corresponding to the proposed excitonic coupling in the ideal Br8-J (left) and Br8-H (right) aggregates. The lowest-lying vibronic states are depicted to map the competing photophysical pathways. Higher-order vibrational states I n the ground electronic state are allowed for both Br8-J and Br8-H; however, the lowest energy vibrational ground state is only allowed for Br8-J, leading the differences in emission wavelength between the two aggregates (the overall energy bandgap is not to scale).

Table S6. Crystallographic packing parameters defining the excitonic coupling in Br8 polymorphs.

Polymorph	***Excitonic Assignment***	***Intermolecular Centroid Distance (R)***	***Kasha Slip Angle (θ)****
Br8-H	H-aggregate	4.69 Å	86.57°
Br8-J	J-aggregate	5.35 Å	44.33°

Table S7. Photoluminescence quantum yield (QY) values for Br8-J and Br8-H polymorphs in microcrystals and thin-film forms.

Polymorph	***Form***	***QY (%)***
Br8-J	Microcrystals	38 (±2)
	Thin film	8 (±0.8)
Br8-H	Microcrystals	2 (±0.5)
	Thin film	0.2 (±1.2)
